# Lipoprotein(a) levels and risk of adverse events after myocardial infarction in patients with and without diabetes

**DOI:** 10.1007/s11239-022-02701-w

**Published:** 2022-09-20

**Authors:** Angelo Silverio, Francesco Paolo Cancro, Marco Di Maio, Michele Bellino, Luca Esposito, Mario Centore, Albino Carrizzo, Paola Di Pietro, Anna Borrelli, Giuseppe De Luca, Carmine Vecchione, Gennaro Galasso

**Affiliations:** 1 Department of Medicine, Surgery and Dentistry, University of Salerno, Baronissi (Salerno), Italy; 2 Vascular Pathophysiology Unit, IRCCS Neuromed, Pozzilli, Isernia Italy; 3 San Giovanni di Dio e Ruggi d’Aragona University Hospital, Salerno, Italy; 4 Clinical and Experimental Cardiology, AOU Sassari, Sassari, Italy; 5 Department of Medicine, Surgery and Dentistry, University of Salerno, University Hospital San Giovanni di Dio e Ruggi d’Aragona, Largo Città di Ippocrate, 84131 Salerno, Italy

**Keywords:** Cholesterol, Mortality, Outcome, Percutaneous coronary intervention, Acute coronary syndrome

## Abstract

**Introduction**: The aim of this study was to evaluate the association of lipoprotein(a) [Lp(a)] levels with long-term outcome in patients with recent history of myocardial infarction (MI), and to investigate if diabetes may influence this association.

**Methods**: Consecutive MI patients who underwent urgent/emergent coronary angiography from February 2013 to June 2019 were prospectively collected. The primary outcome was the composite of MI recurrence and all-cause death. The propensity score weighting technique was used to account for covariates potentially influencing the relationship between Lp(a) levels and the study outcomes.

**Results**: The study population consisted of 1018 post-MI patients (median age 63 years). Diabetes was reported in 280 patients (27.5%), who showed lower Lp(a) levels than patients without diabetes (p = 0.026). At a median follow-up of 1121 days, the primary outcome was reported in 182 patients (17.9%). At univariable Cox regression analysis, Lp(a) was associated with the risk of the primary outcome in the overall population and in non-diabetic patients, but not in diabetics. The adjusted Cox regression analysis confirmed the independent association between Lp(a) values and the primary outcome in non-diabetic patients, but not in diabetics.

Lp(a) levels > 70 mg/dL were independently associated with the risk of the primary outcome in non-diabetic patients (adjusted HR: 2.839; 95% CI, 1.382–5.832), but not in diabetics.

**Conclusions**: In this real-world post-MI population, increasing Lp(a) levels were significantly associated with the risk of recurrent MI and all-cause death, and very high Lp(a) serum concentration independently predicted long-term outcome in non-diabetic patients, but not in diabetics.

**Supplementary Information:**

The online version contains supplementary material available at 10.1007/s11239-022-02701-w.

## Introduction

Lipoprotein(a) [Lp(a)] is a plasma lipoprotein composed by a low-density lipoprotein (LDL) and a molecule of apoliprotein B100 covalently bound, via disulfide bonds, to a plasminogen-like particle named apolipoprotein(a) [Apo(a)].[1] Lp(a) is an emerging risk factor for atherosclerotic cardiovascular disease (ASCVD), which may influence patient’s outcome independently of plasma LDL and high-density lipoprotein cholesterol (HDL-C) levels.[2, 3] Recent studies showed a positive continuous association between Lp(a) levels and the probability of recurrent ischemic events in patients who experienced an acute coronary syndrome (ACS), suggesting its potential for modifying the individual’s residual risk after the acute phase.[4–7].

Diabetes mellitus is a key player in the development and progression of ASCVD, and diabetic patients have a significantly higher risk of adverse cardiovascular and cerebrovascular events (MACCE) compared with non-diabetic subjects.[8] This evidence was confirmed in patients with history of prior myocardial infarction (MI), who are exposed to a higher risk of recurrent ischemic events when diabetes coexists.[9].

Previous studies have investigated the contribution of Lp(a) as risk factor in patients and without diabetes, but this evidence is limited to the general population or to patients with chronic coronary syndrome (CCS).[10–13] ACS patients have a higher risk profile, with an estimated MACCE incidence at 1 year of about 20%. Also, of those who were event free during the first year after MI, 1 in 5 experience an adverse event during the following 3 years.[14] The prognostic significance of the association between high Lp(a) levels and diabetes has never been investigated in this very-high risk population.

The aim of this study was to evaluate the effect of Lp(a) serum levels on long-term clinical outcome of post-MI patients, and to investigate if diabetes may influence the association between Lp(a) levels and the risk adverse events in this patients’ population.

## METHODS

### Study population

This was an observational, retrospective, single-centre, cohort study including patients with MI diagnosis admitted at the University Hospital of Salerno (Italy) from February 2013 to June 2019 and discharged to home or rehabilitation facilities. All consecutive patients with MI who underwent urgent/emergent coronary angiography during the study period were prospectively collected in the Institutional ACS registry. MI was defined, according to the Fourth Universal Definition, as the rise and/or fall of troponin values with at least one value above the 99th percentile upper reference limit and with at least one of the followings: symptoms of acute myocardial ischemia; new ischemic ECG changes; development of pathological Q waves; imaging evidence of new loss of viable myocardium or new regional wall motion abnormality in a pattern consistent with an ischemic etiology; identification of a coronary thrombus by angiography including intracoronary imaging or by autopsy.[15].

The study included patients who underwent percutaneous coronary intervention (PCI) and/or coronary artery bypass grafting (CABG); patients who underwent conservative treatment were excluded. Only drug-eluting stents were implanted during the study period. The availability of Lp(a) serum level and clinical outcome information was considered as an inclusion criterion for this study.

For the purpose of the present analysis, patients were divided into two groups according to the history of diabetes, defined as documented history of diabetes in patients’ medical records and/or current treatment with glucose-lowering agents at the time of the hospitalization.

#### Informed consent

was obtained from all individual participants included in the study. The study was approved by the local ethics committee. The investigation conforms to the principles outlined in the Declaration of Helsinki.

### Data collection

During the hospitalization, demographic, clinical, laboratory, echocardiographic, angiographic, and PCI procedural data were collected.

Blood samples were collected in all patients at admission to determine hemoglobin, peak troponin, and creatinine serum levels. Glomerular filtration rate (GFR) was estimated by using the Chronic Kidney Disease Epidemiology Collaboration equation. After 24 h from admission, serum levels of total cholesterol, HDL-C, LDL-C, triglyceride, and Lp(a) were systematically determined.

Echocardiography was performed in all patients at admission. Coronary angiography and procedural data were also systematically collected.

### Follow-up and outcome measures

Follow-up data were obtained through outpatient clinic visits, medical records of new hospitalizations, or telephone interview. For some deceased patients, the information were obtained by telephone interview of the treating physicians or the next of kin.

In this study, the clinical outcome was assessed at the longest available follow-up. The primary outcome was the composite of recurrent MI and all-cause death. Secondary outcome measures included the single components of the primary outcome. Fatal and non-fatal recurrent MI was defined by the presence of angina symptoms with typical ECG changes and elevated cardiac troponin levels with at least one value above the 99th percentile upper reference limit. The secondary outcome measure was the occurrence of death for any cause.

### Statistical analysis

The distribution of continuous data was tested with the Kolmogorov-Smirnov and the Shapiro-Wilk test. Normally distributed variables were expressed as mean ± standard deviation, whereas non-normal ones as median and interquartile range. Categorical variables were reported as numbers and percentages. Continuous normally distributed variables were compared by using the Student t test, whereas differences between non-normally distributed variables were tested with Mann-Withney U test. Categorical variables were compared with chi-squared test, or Fisher exact test when appropriate. Lp(a) serum concentrations was expressed for increasing range values (≤ 10, > 10–30, > 30–50, > 50–70, and ≥ 70 mg/dL). The association between Lp(a) range values and the risk of the study outcomes was assessed by using Cox proportional hazard regression model. The unadjusted and adjusted risk for the outcomes of interest was calculated using the lowest category as reference, and the association across Lp(a) range ordered groups was expressed as p_trend_. The unadjusted and adjusted risk for the outcomes of interest was also calculated using Lp(a) serum concentrations as continuous value. The results of the Cox analyses were presented as hazard ratio (HR) with their 95% confidence intervals (CI). The hypothesis of a divergent effect of Lp(a) levels on the study outcomes in diabetic and non-diabetic patients was tested and p value for interaction were calculated.

The propensity score weighting technique was used to account for multiple conditions potentially affecting the association between Lp(a) and the study outcomes.[16] The propensity score model was developed using a non-parsimonious approach and by incorporating a large number of baseline covariates potentially related to the exposure [Lp(a) levels] and or the study outcomes regardless of their statistical significance or collinearity with other variables included in the model. The following baseline covariates were entered in the propensity score model: age, sex, hypertension, hyperlipidemia, smoking habits, diabetes, history of coronary artery disease (CAD), obesity, clinical presentation as STEMI, Lp(a) serum levels, GFR at admission, total cholesterol, HDL-C LDL-C, multivessel disease, treated vessel by PCI, and coronary artery by-pass grafting. Diabetes was excluded from the propensity score model in the Cox analysis of subgroups with and without diabetes.

After weighting, a standardized mean difference below 0.10, which reflects an optimal balance, was achieved for all covariates included in the propensity score model.

The rate of missing baseline values was minimal in the dataset and reported in eTable 1. Missing data, if any, were handled using multiple imputations with the method of chained equations. For all test, a p value < 0.05 was considered statistically significant. Statistical analysis was performed by using SPSS version 25.0 (SPSS Inc., Chicago, Illinois) and R version 3.5.1 (R Foundation for Statistical Computing, Vienna, Austria).

## RESULTS

### Study population

Of 1,060 patients, in-hospital death occurred in 42 patients (4.0%). Clinical outcome information was available in all-cases; thus, the final study population consisted of 1,018 patients who were discharged to home or rehabilitation facilities. The baseline characteristics of the study population are summarized in Table 1. Diabetes was reported in 280 cases (27.5%). The median age was 63 years (53–77) and patients with diabetes were significantly older than those without (p < 0.001). Expectedly, patients with diabetes showed a different clinical profile characterized by higher prevalence of hypertension (p < 0.001), hyperlipidemia (p < 0.001), obesity (p < 0.001), prior MI (p < 0.001), and prior PCI (p < 0.001). Noteworthy, diabetic patients were less often active smokers than non-diabetic patients. Non-ST elevation MI (NSTEMI) as clinical presentation was prevalent among diabetic patients; conversely, ST-elevation MI (STEMI) was more common in non-diabetic patients (p = 0.003).

**Table 1:** Baseline characteristics of the study population

Variable	Overall population (N = 1018)	Patients with diabetes (N = 280)	Patients without diabetes (N = 738)	p-value
Age (years)	63 (54.0–73.0)	68.0 (59–77)	61.0 (51–72)	< 0.001
Men, N (%)	771 (75.7)	213 (76.1)	558 (75.6)	0.878
Hypertension, N (%)	668 (65.7)	218 (77.9)	450 (61.1)	< 0.001
Hyperlipidemia, N (%)	481 (47.3)	158 (56.4)	323 (43.9)	< 0.001
Active smokers, N (%)	506 (49.7)	109 (39.1)	397 (53.9)	< 0.001
Obesity, N (%)	258 (25.4)	106 (38.0)	152 (20.7)	< 0.001
History of CAD, N (%)	191 (18.8)	88 (31.4)	103 (14.0)	< 0.001
Prior MI, N (%)	140 (13.8)	62 (22.1)	78 (10.6)	< 0.001
Prior PCI, N (%)	129 (12.7)	61 (21.6)	68 (9.2)	< 0.001
Clinical presentation, N (%)				
STEMI	771 (75.7)	195 (69.6)	579 (78.5)	0.003
NSTEMI	247 (24.3)	85 (30.4)	159 (21.5)	0.003
LVEF (%), N (%)				< 0.001
< 35	69 (6.9)	28 (10.1)	41 (5.7)	
35–45	260 (26.0)	91 (32.7)	168 (23.4)	
45–55	292 (29.3)	77 (27.7)	215 (29.9)	
> 55	377 (37.8)	82 (29.5)	295 (41.0)	
Hemoglobin (g/dL)	14.3 (13.0-15.5)	13.8 (12.4–15.3)	14.5 (13.3–15.6)	< 0.001
eGFR (mL/min)	80 (60.0–94.0)	75.0 (51.3–92.0)	82.0 (64.0–95.0)	< 0.001
Peak troponin (pg/mL)	14.0 (3.3–56.2)	14.4 (2.7–58.3)	14.0 (3.5–55.4)	0.997
Total cholesterol (mg/dL)	180 (147–211)	157 (128–193)	184 (155–215)	< 0.001
HDL-Cholesterol (mg/dL)	43 (81–135)	42 (34–49)	44 (37–54)	0.002
LDL-Cholesterol (mg/dL)	107 (36–52)	89 (64–122)	114 (87–137)	< 0.001
Triglycerides (mg/dL)	116 (84–162)	115 (84–167)	117 (83–158)	0.900
Lipoprotein(a) (mg/dl)	10 (10–30)	10 (10–30)	20 (10–40)	0.026
≤ 10	520 (51.1)	159 (56.8)	361 (48.9)	0.025
11–30	260 (25.5)	70 (25.0)	190 (25.7)	0.808
31–50	108 (10.6)	18 (6.4)	90 (12.2)	0.008
51–70	56 (5.5)	14 (5.0)	42 (5.7)	0.666
> 70	74 (7.3)	19 (6.8)	55 (7.5)	0.714
SYNTAX Score	14 (8.0-22.5)	18.0 (10–26)	12.0 (7–22)	< 0.001
Multivessel coronary disease, N (%)	413 (40.6)	153 (54.6)	260 (35.2)	0.001
Treated coronary artery by PCI, N (%)				
Left main	15 (1.5)	8 (2.9)	7 (1.0)	0.025
Left anterior descending	473 (46.5)	131 (46.8)	342 (46.3)	0.899
Left circumflex	177 (17.4)	51 (18.2)	126 (17.1)	0.668
Right coronary artery	303 (29.8)	78 (27.9)	225 (30.5)	0.412
CABG	80 (7.9)	33 (11.8)	47 (6.4)	0.004

Continuous normally distributed variables are expressed as mean ± SD. Categorical variables are expressed as N (%). Continuous non-normally distributed variables are expressed as median (interquartile range). Hyperlipidemia was defined by laboratory data showing LDL-C > 160 mg/dl, HDL-C < 40 mg/dl in men or < 50 mg/dl in women, fasting triglycerides > 150 mg/dl, clinical diagnosis of primary hyperlipidemia, or previous lipid lowering therapy. History of CAD was defined as prior acute coronary syndrome, coronary revascularization, or established CAD. Obesity was defined by body mass index value ≥ 30 kg/m^2^

CABG, coronary artery bypass graft; CAD, coronary artery disease; eGFR, estimated glomerular filtration rate; HDL-C, high-density lipoprotein cholesterol; LDL-C, low-density lipoprotein cholesterol; LVEF, left ventricular ejection fraction; MI, myocardial infarction; PCI, percutaneous coronary intervention; TIMI, Thrombolysis in Myocardial Infarction

The median value of Lp(a) plasma levels was 10 mg/dL, and patients with diabetes showed significantly lower levels of Lp(a) than patients without diabetes (p = 0.025).

Diabetic patients showed higher SYNTAX score value (p < 0.001), and underwent more frequently to left main PCI (p = 0.025) and CABG (p = 0.004).

### Long-term outcomes

At a median follow-up of 1121 (763–1387) days, the primary outcome was reported in 182 patients (17.9%), recurrent MI in 109 (10.7%), and all-cause death in 100 (9.8%).

**Fig. 1:**
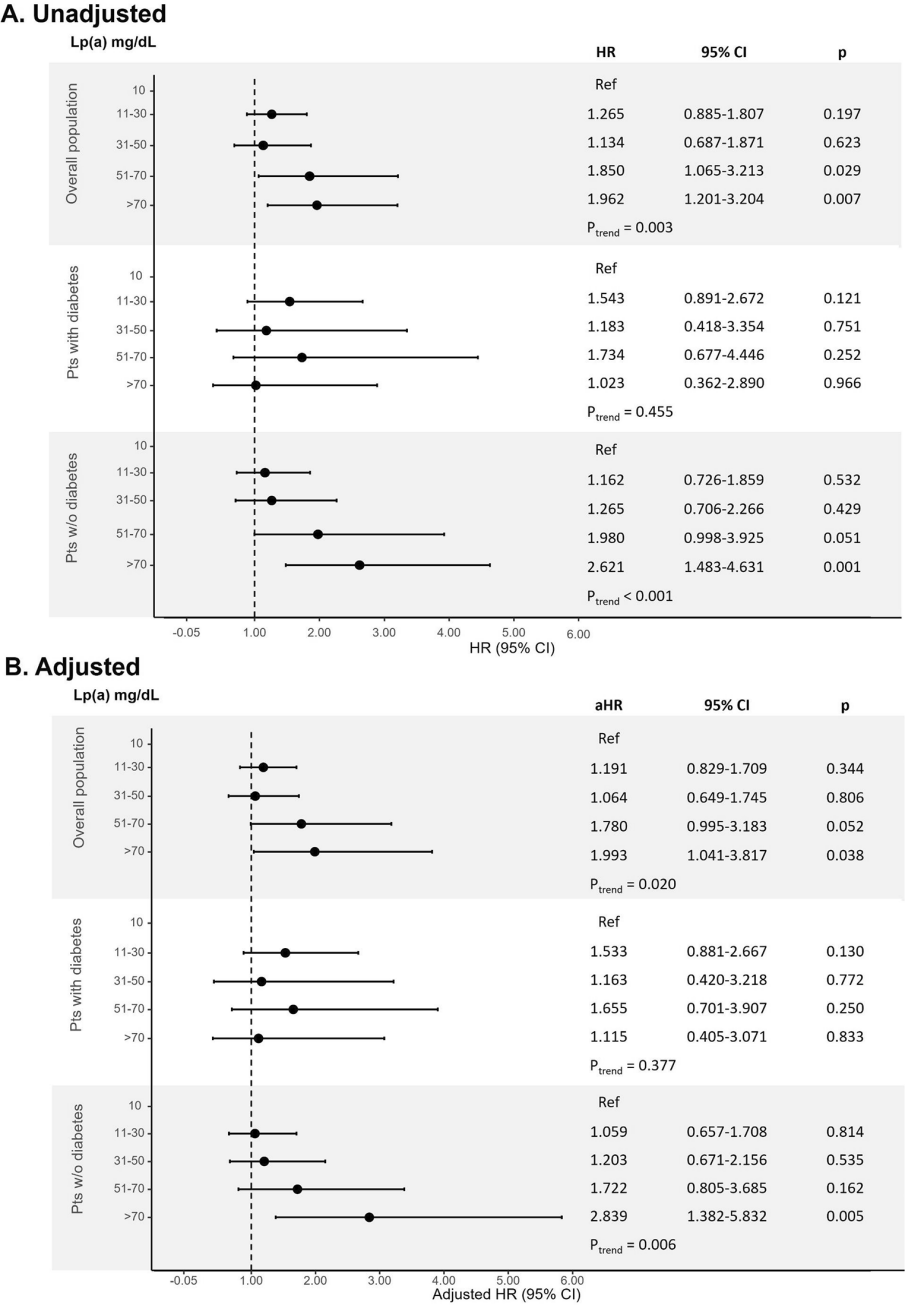
**Unadjusted (panel A) and adjusted (panel B) analysis for the risk of the primary outcome according to Lp(a) ordered groups in the overall population and in patients with and without diabetes** Cox proportional-hazards regression model for the risk of the composite of recurrent MI and all-cause death; the HR were calculated for Lp(a) range categories with the lowest category (≤ 10 mg/dL) as reference aHR, adjusted hazard ratio; CI, confidence interval; HR, hazard ratio; Lp(a), lipoprotein(a)

At unadjusted Cox regression analysis, there was a statistical increasing risk for the primary outcome across Lp(a) range ordered groups (p_trend_ = 0.003; Fig. 1). Compared with the lowest Lp(a) category (≤ 10 mg/dL), patients with Lp(a) level of 51–70 (HR: 1.850; 95% CI, 1.065–3.213) and > 70 mg/dL (HR: 1.962; 95% CI, 1.201–3.204) showed a significantly higher risk for the composite outcome.

The association with the risk for the primary outcome was confirmed across Lp(a) range groups in patients without diabetes (p_trend_ < 0.001), but not in those with diabetes (p_trend_ = 0.455). Compared with the lowest Lp(a) category, non-diabetic patients with Lp(a) level > 70 mg/dL (HR: 2.621; 95% CI, 1.483–4.631) showed a significantly higher risk for the composite outcome.

After propensity score weighting, a significant association between increasing Lp(a) range values and the primary outcome was confirmed in the overall population (p_trend_ = 0.020) and in the non-diabetic group (p_trend_ = 0.006; Fig. 1). Also, compared with the lowest Lp(a) category, non-diabetic patients with very high Lp(a) levels > 70 mg/dL showed a significantly higher risk of the composite outcome. (adjusted HR: 2.839; 95% CI, 1.382–5.832).

These results were confirmed by the analysis of Lp(a) continuous value both at univariable and at adjusted Cox regression models (Table 2).

**Table 2:** Unadjusted and adjusted analysis for the association of Lp(a) levels with risk of the study outcomes

Primary composite outcome								
	HR	95% CI	p	p for interaction	Adjusted HR	95% CI	p	p for interaction
Overall population	2.134	1.380–3.300	0.001	0.016	1.634	0.896–2.980	0.109	0.042
Patients with diabetes	1.317	0.571–3.035	0.518		1.466	0.732–2.937	0.280	
Patients w/o diabetes	2.731	1.655–4.507	< 0.001		2.119	1.087–4.131	0.028	
**Myocardial infarction**								
	HR	95% CI	p	p for interaction	Adjusted HR	95% CI	p	p for interaction
Overall population	2.421	1.406–4.170	0.001	0.029	2.057	1.015–4.172	0.046	0.134
Patients with diabetes	1.690	0.661–4.316	0.273		2.003	0.962–1.169	0.063	
Patients w/o diabetes	3.104	1.619–5.951	0.001		2.403	1.119–5.162	0.025	
**All-cause death**								
	HR	95% CI	p	P for interaction	Adjusted HR	95% CI	p	p for interaction
Overall population	1.937	1.076–3.486	0.027	0.038	1.249	0.636–2.454	0.519	0.094
Patients with diabetes	0.935	0.265–3.294	0.916		0.728	0.228–2.321	0.591	
Patients w/o diabetes	2.629	1.371–5.039	0.004		1.936	0.915–4.099	0.084	

Cox proportional-hazards regression model for the risk of the association between Lp(a) and the study outcomes; the HR were calculated for Lp(a) continuous values. CI, confidence interval; HR, hazard ratio; Lp(a), lipoprotein(a)

**Fig. 2:**
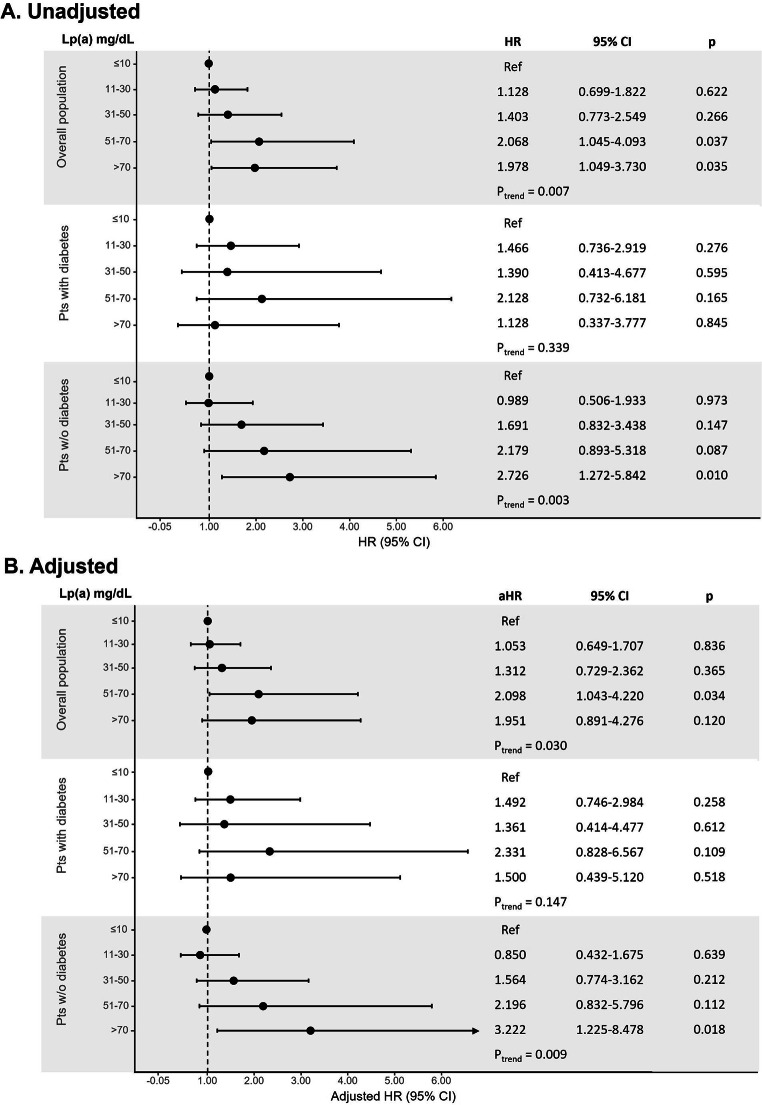
**Unadjusted (panel A) and adjusted (panel B) analysis for the risk of MI recurrence according to Lp(a) ordered groups in the overall population and in patients with and without diabetes** Cox proportional-hazards regression model for the risk of recurrent MI; the HR were calculated for Lp(a) range categories with the lowest category (≤ 10 mg/dL) as reference aHR, adjusted hazard ratio; CI, confidence interval; HR, hazard ratio; Lp(a), lipoprotein(a)

**Fig. 3:**
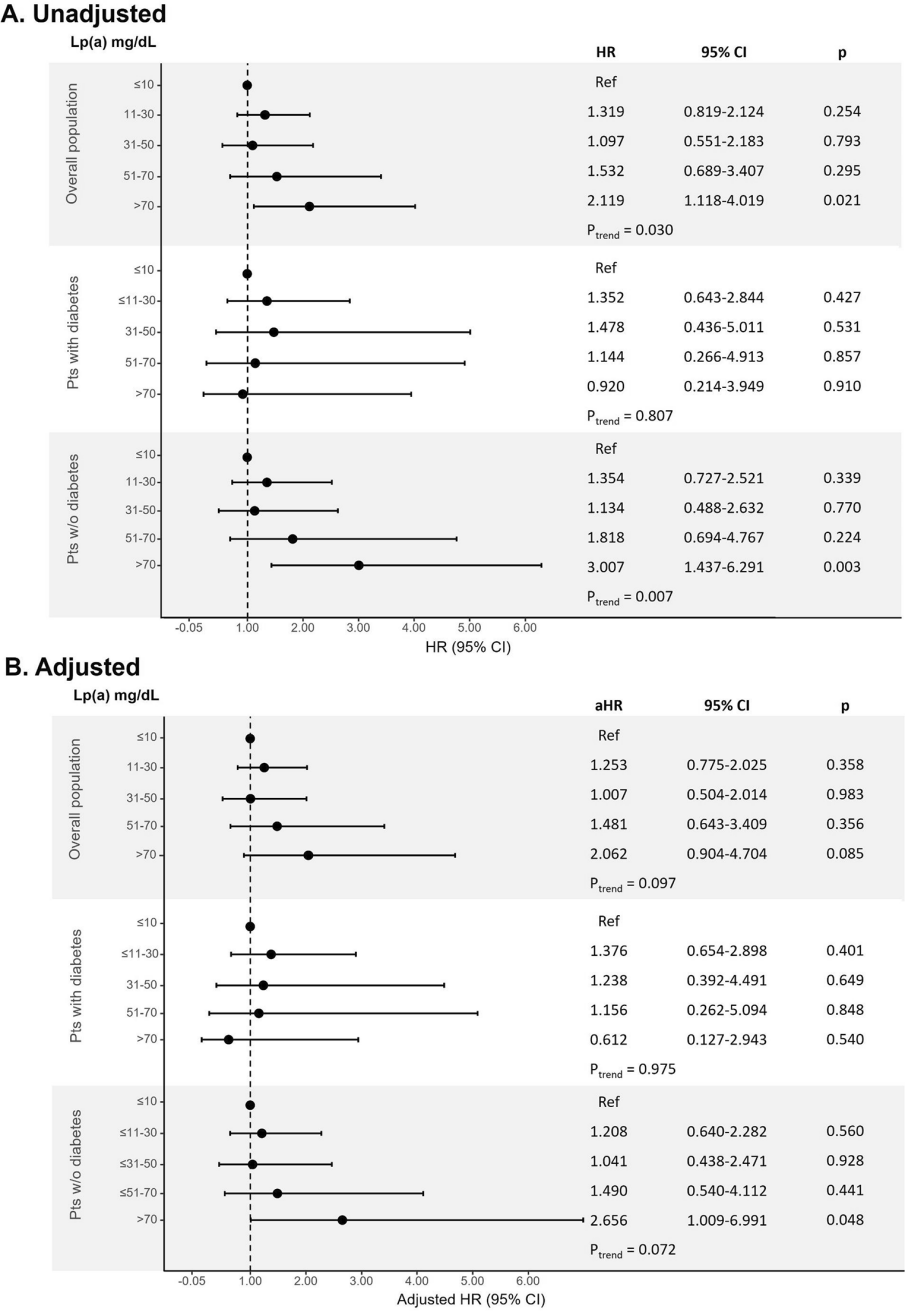
**Unadjusted (panel A) and adjusted (panel B) analysis for the risk of all-cause death according to Lp(a) ordered groups in the overall population and in patients with and without diabetes** Cox proportional-hazards regression model for the risk of all-cause death; the HR were calculated for Lp(a) range categories with the lowest category (≤ 10 mg/dL) as reference aHR, adjusted hazard ratio; CI, confidence interval; HR, hazard ratio; Lp(a), lipoprotein(a)

At univariable analysis, there was a statistical increasing risk for the secondary outcomes across Lp(a) range groups in the overall population and in patients without diabetes, but not in diabetics (Figs. 2 and 3). After propensity score weighting, non-diabetic patients with very high Lp(a) > 70 mg/dL showed a significantly higher risk of recurrent MI (HR: 3.222; 95% CI, 1.225–8.478; Fig. 2) and all-cause mortality (HR: 2.656; 95% CI, 1.009–6.991; Fig. 3).

## DISCUSSION

The main findings of the present real-world study including a contemporary cohort of MI patients, can be summarized as follows: (1) patients with diabetes accounted for 28% of cases and showed lower Lp(a) levels than patients without diabetes; (2) increasing values of Lp(a) serum levels were significantly associated with the risk of recurrent MI and all-cause death in the overall population; this association was confirmed among non-diabetic patients, but not in diabetics; (3) very high Lp(a) levels (> 70 mg/dL) were independently associated with the risk of death and recurrent MI in non-diabetic patients; this association was not confirmed in diabetic patients.

In this post-MI population, we reported a prevalence of diabetes of 28%, which is consistent with previous multicenter registries. [14] In these patients, Lp(a) levels were lower compared to patients without diabetes, confirming the results of previous observational reports. [17–19] Recent studies also reported an inverse association between Lp(a) levels and the percentage of incident diabetes mellitus. [18, 20] Although the pathophysiological basis of this inverse correlation is not completely clarified, a negative association between serum concentrations of insulin and Lp(a) has been described, suggesting a role of insulin in determining the levels of Lp(a). [21, 22] Marzano et al., in a cohort of 527 non-diabetic hypertensive patients, showed that insulin resistance and higher fasting insulin levels correlated with lower Lp(a) levels.[23] In addition, Neele et al. reported a dose-dependent inhibitory effect of high insulin plasma concentration on the hepatic synthesis of Apo(a).[24].

Lp(a) may increase the probability of adverse cardiovascular events and mortality after MI through different mechanisms: (1) Lp(a) particles promote the atherogenesis by crossing the endothelial barrier and the arterial intima, and delivering cholesterol like LDL to the plaque; (2) promotion of the adhesion and migration of monocytes through the interaction of the Apo(a) moiety with the β2-integrin Mac-1; (3) pro-thrombotic effect due to the similarity of Apo(a) with plasminogen, resulting into a competitive inhibition of plasmin generation.[25].

The role of Lp(a) for risk stratification of patients with established ASCVD has long been debated but recent studies reported a significant association between Lp(a) levels and the risk of recurrent ischemic events after MI. [4, 6, 7] In the present study, we confirmed the independent association between increasing Lp(a) levels and adverse events in post-MI patients, and we found that diabetes played as a modifier of the effect of Lp(a) on long-term clinical outcome.

Jin and colleagues, in a multicenter registry including 5143 patients with CCS, reported a significant association between elevated Lp(a) and the risk of adverse cardiovascular events in both non-diabetic patients and in those with pre-diabetes or diabetes[10]. However, they did not include patients with ACS and reported 19% of non-diabetic patients, which is significantly lower than in our study.

Konishi et al., in a cohort 1136 diabetic patients treated with PCI (25% of ACS), reported no significant association between Lp(a) levels and all-cause mortality, which is consistent with our study [12]. Also, Saely et al., in a prospective cohort of 587 patients undergoing coronary angiography (23% diabetics), reported lower Lp(a) plasma levels in diabetics compared with non-diabetic patients. In this study, Lp(a) also emerged as an independent predictor of adverse cardiovascular events only in non-diabetics, which is consistent with our study [11].

Diabetes is an established risk factor for ASCVD and is frequently associated with other metabolic disorders such as dyslipidemia and insulin resistance [26]. Alone, or in combination with these conditions, diabetes impairs the endothelial function and promotes the onset and rapid progression of atherosclerosis [27]. In diabetic patients experiencing MI, we can assume that diabetes is closely involved in the development and progression of atherosclerotic plaques. Conversely, in patients who do not have diabetes, other risk factors are involved in the pathogenesis of CAD and may influence the natural history of patients after the first MI event.

Diabetic patients, besides the lower Lp(a) plasma levels, have Lp(a) particles with larger Apo(a) isoforms compared to non-diabetic subjects.[3, 17–19] These larger isoforms of Apo(a) tend to aggregate and to be degraded into the hepatic cells [19] and seem to play a lower atherogenic effect compared with Lp(a) particles with smaller Apo(a) isoforms.[25, 28, 29] This evidence was confirmed by a metanalysis including 40 studies, which showed that smaller isoforms of Apo(a) had an approximately 2-fold higher probability of CAD and/or ischemic stroke than those with larger isoforms.[30] This association is still poorly explained in a mechanistically way, but smaller isoforms of Apo(a) seem to have a higher affinity in binding fibrin and inhibiting plasminogen activation, with a consequent mitigation of fibrinolysis processes and amplification of the thrombotic effect of Lp(a).[31, 32].

Lp(a) serum concentration is largely genetically determined, with a negligible influence of diet, environment, and physical exercise [33]. To date, there is no approved pharmacological therapy for treatment of elevated Lp(a), and lipid-lowering drugs commonly used, including statins and ezetimibe, have no effect on its serum levels. More recently, subanalyses from randomized studies showed a significant reduction of Lp(a) levels and of the risk of adverse events in patients treated with PCSK9 inhibitors vs. placebo [5, 34, 35]. Novel molecules have the potential of reducing Lp(a) levels, but their safety and efficacy need to be tested in randomized controlled trials [36].

Our study highlights the strong need for a pharmacological therapy targeting Lp(a) plasma levels, particularly in populations where Lp(a) has a greater prognostic significance, such as post-MI patients without diabetes.

## STUDY LIMITATIONS

The results of this study need to be interpreted considering some limitations. First, the retrospective, single-center, design and the relatively small sample size of the study.

Second, we did not provide data on lipid control during the follow-up. However, lipid-lowering drugs are generally ineffective in reducing serum Lp(a) concentrations, with the exception of PCSK9 inhibitors used in a small number of patients, and a single measurement of Lp(a) concentration was proven to be sufficient for informing cardiovascular risk in large patient cohorts[37].

Third, we did not report the Lp(a) values during follow-up. Since Lp(a) may act as an acute-phase protein, its levels may increase during the acute phase and remain high for several weeks after an ACS.

Lastly, the relationship between two variables may be different in selected clinical settings (MI patients in this study) as a consequence of a distortion effect, and not only because of restriction of range. Therefore, our results cannot be extended to the general population and should be carefully considered as hypothesis-generating.

## Conclusion

In this real-world population of stable post-MI patients, Lp(a) serum levels were lower in diabetic than in non-diabetic patients. Increasing Lp(a) levels were significantly associated with the risk of the composite of recurrent MI and all-cause death, and serum concentration > 70 mg/dL independently predicted the risk of adverse cardiovascular outcome, but only in patients without diabetes.

These results reinforce the importance of routine assessment of Lp(a) levels after MI, particularly in patients without diabetes.

## Electronic supplementary material

Below is the link to the electronic supplementary material.

Supplementary Material 1
