# Supplementary material for: Lipoprotein(a) levels and risk of adverse events after myocardial infarction in patients with and without diabetes
Source: J Thromb Thrombolysis. 2022 Sep 20;54(3):382–92. doi: 10.1007/s11239-022-02701-w (PMC9553824; doi:10.1007/s11239-022-02701-w)
Supplement: Supplementary file 1 — Supplementary Material 1 [file 11239_2022_2701_MOESM1_ESM.docx]

SUPPLEMENTAL MATERIALS

Lipoprotein(a) levels and risk of adverse events after myocardial infarction in patients with and without diabetes

Angelo Silverio, MD, Francesco Paolo Cancro, MD, Marco Di Maio, MD, Michele Bellino, MD, Luca Esposito, MD, Mario Centore, MD, Albino Carrizzo, PhD, Paola Di Pietro, PhD, Anna Borrelli, MD, Giuseppe De Luca, MD, PhD, Carmine Vecchione, MD, Gennaro Galasso, MD, PhD.

**eTable 1.** **Rates of missing baseline values**

| **Variable** | **Overall population**  **(N = 1018)** |
| --- | --- |
| Age (years), N (%) | 0 |
| Men, N (%) | 0 |
| Hypertension, N (%) | 2 (0.2) |
| Hyperlipidemia, N (%) | 2 (0.2) |
| Active smokers, N (%) | 3 (0.3) |
| Obesity, N (%) | 3 (0.3) |
| History of CAD, N (%) | 2 (0.2) |
| Prior MI, N (%) | 0 |
| Prior PCI, N (%) | 0 |
| Clinical presentation, N (%) | 0 |
| LVEF (%), N (%) | 21 (2.1) |
| Hemoglobin (g/dL), N (%) | 53 (5.2) |
| eGFR (mL/min), N (%) | 27 (2.7) |
| Peak troponin (pg/mL), N (%) | 29 (2.9) |
| Total cholesterol (mg/dL), N (%) | 0 |
| HDL-Cholesterol (mg/dL), N (%) | 0 |
| LDL-Cholesterol (mg/dL), N (%) | 0 |
| Triglycerides (mg/dL), N (%) | 0 |
| Lipoprotein(a) (mg/dl), N (%) | 0 |
| SYNTAX Score, N (%) | 27 (2.7) |
| Multivessel coronary disease, N (%) | 0 |
| Treated coronary artery by PCI, N (%) | 0 |
| CABG | 0 |

CABG, coronary artery bypass graft; CAD, coronary artery disease; eGFR, estimated glomerular filtration rate; HDL-C, high-density lipoprotein cholesterol; LDL-C, low-density lipoprotein cholesterol; LVEF, left ventricular ejection fraction; MI, myocardial infarction; PCI, percutaneous coronary intervention; TIMI, Thrombolysis in Myocardial Infarction.
